# Ogilvie’s syndrome—is there a cutoff diameter to proceed with upfront surgery?

**DOI:** 10.1007/s00423-021-02407-2

**Published:** 2022-01-12

**Authors:** Katharina Joechle, Jessica Guenzle, Stefan Utzolino, Stefan Fichtner-Feigl, Lampros Kousoulas

**Affiliations:** Department of General and Visceral Surgery, Medical Center-University of Freiburg, Faculty of Medicine, University of Freiburg, Hugstetterstr. 55, 79106 Freiburg, Germany

**Keywords:** Ogilvie, Ogilvie’s syndrome, Acute colonic pseudo-obstruction, Colonic dilatation

## Abstract

**Purpose:**

Although Ogilvie’s syndrome was first described about 70 years ago, its etiology and pathogenesis are still not fully understood. But more importantly, it is also not clear when to approach which therapeutic strategy.

**Methods:**

Patients who were diagnosed with Ogilvie’s syndrome at our institution in a 17-year time period (2002–2019) were included and retrospectively evaluated regarding different therapeutical strategies: conservative, endoscopic, or surgical.

**Results:**

The study included 71 patients with 21 patients undergoing conservative therapy, 25 patients undergoing endoscopic therapy, and 25 patients undergoing surgery. However, 38% of patients (*n* = 8) who were primarily addressed for conservative management failed and had to undergo endoscopy or even surgery. Similarly, 8 patients (32%) with primarily endoscopic treatment had to proceed for surgery. In logistic regression analysis, only a colon diameter ≥ 11 cm (*p* = 0.01) could predict a lack of therapeutic success by endoscopic treatment. Ninety-day mortality and overall survival were comparable between the groups.

**Conclusion:**

As conservative and endoscopic management fail in about one-third of patients, a cutoff diameter ≥ 11 cm may be an adequate parameter to evaluate surgical therapy.

## Introduction

The first cases of acute colonic pseudo-obstruction were described in 1948 by Sir Heneage Ogilvie [1], therefore known as Ogilvie’s syndrome. It is defined as dilatation of a part or all of the colon without any mechanical obstruction or underlying organic disease such as acute colitis [2]. Although the pathophysiology is still not fully understood and multiple theories are discussed including a denervation of the autonomic nervous system, a vascular and hormonal impact, and metabolic, pharmalogical, and infectious reasons [2], it is notable that it mainly occurs in hospitalized patients who are in about one-fifth treated following childbirth, pelvic/orthopedic surgery, or spinal cord trauma [3].

Not only the pathophysiology of Ogilvie’s syndrome remains unclear but also the optimal treatment approach. There are different strategies including restrictive management addressing potentially underlying conditions such as electrolyte repletion or termination of narcotics and opioids [2, 3] and placing a nasogastric tube for gastrointestinal decompression. Even if this management is recommended for 48–72 h after diagnosis of Ogilvie’s syndrome by the guidelines of the American Society for Gastrointestinal Endoscopy [4], the severe risk of colonic perforation or ischemia must be always reminded, increasing by increased colon diameter [2] and leading to a high mortality rate of about 40–50% [5]. Therefore, it is crucial to identify the correct time point to approach the adequate next therapeutic step to lower morbidity and mortality associated with Ogilvie’s syndrome. In this context, we aimed to identify factors predicting a successful outcome after the different therapeutic strategies.

## Material and methods

### Study population

After obtaining permission from the institutional ethics committee (protocol number 339/20), we retrospectively identified patients who were diagnosed and treated for Ogilvie’s syndrome at our institution between 2002 and 2019 by searching for the ICD10 code K56.0. As this code not only includes Ogilvie’s syndrome but also paralysis or paralytic ileus, these patients were checked by the authors to fulfill the criteria of Ogilvie’s syndrome. Patients with primary Ogilvie’s syndrome as well as patients who developed Ogilvie’s syndrome postoperatively were included in this study.

Patients were divided into three groups regarding the treatment strategy they received: first, conservative treatment including proximal gastrointestinal decompression via nasogastric tube, intravenous fluid, and electrolyte repletion and pharmacologic therapy to restrain bowel motility (neostigmine, Gastrografine, erythromycin, etc.); second, endoscopic treatment with or without placing of a decompression tube; and third, surgical therapy including surgical decompression with and without colonic resection.

Therapeutic failure was defined as being in need to undergo endoscopy or/and surgery for the conservatively treated patients and being in need to undergo surgery for the endoscopically treated patients.

### Definitions

Ogilvie’s syndrome was defined as dilatation of a part or all of the colon without any mechanical obstruction or underlying organic disease such as acute colitis. The diameter of colonic dilatation was measured on computer tomography (CT) scans for the majority of patients (80%). For the other 20% of patients who did not have a CT scan available, an abdominal X-ray was used for the measurement of colonic dilatation in a standardized procedure.

If the initially chosen treatment strategy was not successful and patients hat to proceed to a more invasive treatment, it was defined as therapeutic failure. Thus, treatment failure for patients with initial conservative treatment strategy included to be in need for undergoing endoscopy and/or even surgery and for patients who were primarily addressed for endoscopy if they were in need for surgery.

### Data collection

The following patient data were recorded and the study reported in line with STROCSS criteria [6]: sex, age, comorbidities, reason for hospital admission, time frame between hospital admission and diagnosis of Ogilvie’s syndrome, blood counts of leukocytes, CRP, procalcitonin and lactate at the time of diagnosis of Ogilvie’s syndrome, colonic dilatation in cm, treatment strategy (conservative vs endoscopic vs surgical), number of endoscopies, placement of a decompression tube, reason for surgery, kind of operation, length of stay on intensive care unit (ICU), readmission on ICU, length of hospital stay, 90-day mortality, and survival data counted from the day of diagnosis of Ogilvie’s syndrome.

### Statistical analysis

Statistical analysis was performed using SPSS statistics version 25 (IBM, Armonk, New York, USA). Continuous variables were analyzed using the Mann–Whitney-*U* test and the Kruskal–Wallis test and expressed as medians and ranges. Categorical variables were analyzed using the *χ*^2^ test or Fisher’s exact test, as appropriate, and expressed as absolute values and percentages. Logistic regression analysis was used to predict factors of therapeutic failure for conservative and endoscopic treatment with a cutoff *p*-value of < 0.05 in univariate logistic regression to include factors into multivariate logistic regression. Overall survival (OS) rates were calculated from the day of diagnosis of Ogilvie’s syndrome, estimated using the Kaplan–Meier method, and compared using log-rank statistics. All tests were two-sided and a *p* value < 0.05 was considered to be statistically significant.

## Results

### Patient characteristics

Between 2002 and 2019, 71 patients were treated for Ogilvie’s syndrome at our institution. Of these, 21 patients (30%) underwent conservative treatment, 25 patients (35%) underwent endoscopic treatment, and 25 patients (35%) underwent upfront surgery. Patient characteristics of the three groups are shown in Table 1. The median age of the cohort was 67 years (18–92) with Ogilvie’s syndrome occurring more often in male than female patients throughout the groups. The primary reasons for hospital admission were similar between the three groups except for abdominal symptoms (*p* = 0.03) which was noticed to occur more often in patients who had to undergo upfront surgery. Primary Ogilvie’s syndrome was noticed in 37 patients (52%) and 34 patients (48%) developed Ogilvie’s syndrome postoperatively. Of those, 12 patients underwent trauma or orthopedic surgery, 16 had abdominal surgery, and 6 patients developed Ogilvie’s syndrome after vascular or cardiac surgery. Liver surgery as a known risk factor for Ogilvie’s syndrome did not occur in our cohort, but in 12 cases patients developed postoperative Ogilvie’s syndrome after elective abdominal surgery and in 4 cases after emergency abdominal surgery (appendicitis, cholecystitis, perforation, and bleeding). Almost all patients (*n* = 65, 92%) had comorbidities with chronic kidney disease significantly differing between the groups (*p* = 0.04) and occurring the most often in patients who underwent endoscopic treatment. Whereas colon dilatation was comparable between the groups (*p* = 0.76), they significantly differed regarding blood levels of CRP (*p* = 0.03), lactate (*p* = 0.04), and leukocytes (*p* = 0.02) at the time of diagnosis. There was also a statistically significant difference regarding the length of hospital stay (*p* = 0.02). However, comparing only endoscopic and surgical treatment, the length of hospital stay was comparable (*p* = 0.43). For length of ICU stay (*p* = 0.21) and readmission rate to ICU (*p* = 0.92), there was no significant difference between the groups as well as for 90-day mortality (*p* = 0.98).

**Table 1:** Patient characteristics

	Total	Conservative therapy	Endoscopic therapy	Operative therapy	*p* value
	*n* = 71 (100%)	*n* = 21 (30%)	*n* = 25 (35%)	*n* = 25 (35%)	
Age in years, median (range)	67 (18–92)	75 (18–92)	64 (34–86)	60 (21–83)	0.20
Gender, *n* (%)					
Male	52 (73)	17 (81)	19 (76)	16 (64)	0.40
Female	19 (27)	4 (19)	6 (24)	9 (36)	
Primary reason for admission, *n* (%)					
Trauma	14 (20)	6 (29)	5 (20)	3 (12)	0.37
Abdominal focus	31 (44)	9 (38)	7 (28)	16 (64)	0.03
Oncologic reason (CTx, etc.)	6 (8)	2 (10)	3 (12)	1 (4)	0.58
Internal medicine	6 (8)	1 (5)	3 (12)	2 (8)	0.68
Cardiac/vascular	11 (16)	3 (14)	6 (24)	2 (8)	0.29
Neurological/psychiatric	3 (4)	1 (5)	1 (4)	1 (4)	0.99
Comorbidities, *n* (%)	65 (92)	20 (95)	23 (92)	22 (88)	0.68
Arterial hypertension	24 (34)	8 (38)	8 (32)	8 (32)	0.88
CAD	13 (18)	5 (24)	5 (20)	3 (12)	0.57
PAD	6 (9)	2 (10)	2 (8)	2 (8)	0.98
Atrial fibrillation	9 (13)	2 (10)	3 (12)	4 (16)	0.80
Stroke	10 (14)	2 (10)	4 (16)	4 (16)	0.77
Cancer	24 (34)	8 (38)	10 (40)	6 (24)	0.43
Previous abdominal surgery	20 (28)	5 (24)	9 (36)	6 (24)	0.56
Chronic kidney disease	12 (17)	2 (10)	8 (32)	2 (8)	0.04
Pulmonary disorders	18 (25)	8 (38)	7 (28)	3 (12)	0.12
Diabetes	11 (16)	2 (10)	3 (12)	6 (24)	0.34
Obesity	10 (14)	3 (14)	3 (12)	4 (16)	0.92
Alcohol abuse	9 (13)	1 (5)	3 (12)	5 (20)	0.30
Psychiatric disorders	21 (30)	9 (43)	4 (16)	8 (32)	0.13
Days from hospital admission to diagnosis, median (range)	3 (0–44)	3 (0–44)	6 (0–21)	3 (0–15)	0.14
Leukocytes at diagnosis, cells/nl, median (range)°	11.2 (2.5–35.6)	9.7 (3.1–26.4)	14.1 (7.5–35.6)	9.8 (2.5–23.9)	0.02
CRP at diagnosis, mg/L, median (range)*	116 (3–495)	27 (3–495)	150 (4–312)	231 (3–495)	0.03
Lactate at diagnosis, mmol/L, median (range)*	1.6 (0.6–10.1)	1.3 (0.6–4.8)	1.6 (0.7–3.8)	2.3 (0.7–10.1)	0.04
Dilatation of colon in cm, median (range) ~	9 (6–15)	9 (7–12)	9 (6–13)	8 (7–15)	0.76
Length of stay on ICU, days, median (range)	5 (0–49)	4 (0–22)	6 (0–49)	7 (1–47)	0.21
Readmission on ICU, *n* (%)	18 (25)	6 (29)	6 (24)	6 (24)	0.92
Length of hospital stay, days, median (range)	26 (4–127)	10 (4–78)	29 (4–71)	27 (7–127)	0.02
90-day mortality, *n* (%)	11 (20)	3 (21)	3 (19)	5 (21)	0.98

*CTx*, chemotherapy; *CAD*, coronary artery disease; *ICU*, intensive care unit; *PAD*, peripheral artery disease; *CRP*, c-reactive protein; #missing for 51 patients, °missing for 6 patients, *missing for 28 patients, ~ missing for 22 patients

### Conservative management

Conservative treatment was successful for 13 patients (62%) and failed for the remaining 8 patients (38%) who subsequently had to undergo endoscopy (*n* = 2, 9.5%), surgery (*n* = 4, 19%), or both endoscopy and surgery (*n* = 2, 9.5%).

Comparing patients with and without successful conservative management (Table 2) showed that therapeutic failure leads to a significantly higher readmission rate in ICU (*p* = 0.01) and a significantly longer hospital stay (*p* = 0.002). Performing a logistic regression analysis did not identify any predictor of therapeutic failure for patients undergoing conservative management (Table 3).

**Table 2:** Characteristics of patients with and without therapeutic success receiving upfront conservative management

	Total	Therapeutic success	No therapeutic success	*p* value
	*n* = 21 (100%)	*n* = 13 (62%)	*n* = 8 (38%)	
Age in years, median (range)	75 (18–92)	77 (29–92)	74 (18–79)	0.44
Gender, *n* (%)				
Male	17 (81)	10 (77)	7 (88)	0.64
Female	4 (19)	3 (23)	1 (12)	
Primary reason for admission, *n* (%)				
Trauma	6 (29)	2 (15)	4 (50)	0.15
Abdominal focus	9 (38)	6 (46)	2 (25)	0.40
Oncologic reason (CTx, etc.)	2 (10)	1 (8)	1 (12)	1.0
Internal medicine	1 (5)	1 (8)	0 (0)	1.0
Cardiac/vascular	3 (14)	2 (15)	1 (12)	1.0
Neurological/psychiatric	1 (5)	1 (8)	0 (0)	1.0
Comorbidities, *n* (%)	20 (95)	13 (100)	7 (88)	0.38
Arterial hypertension	8 (38)	5 (38)	3 (38)	1.0
CAD	5 (24)	2 (15)	3 (38)	0.33
PAD	2 (10)	0 (0)	2 (25)	0.13
Atrial fibrillation	2 (10)	1 (8)	1 (12)	1.0
Stroke	2 (10)	2 (15)	0 (0)	0.51
Cancer	8 (38)	4 (31)	4 (50)	0.65
Previous abdominal surgery	5 (24)	3 (23)	2 (25)	1.0
Chronic kidney disease	2 (10)	0 (0)	2 (25)	0.13
Pulmonary disorders	8 (38)	5 (38)	3 (38)	1.0
Diabetes	2 (10)	1 (8)	1 (12)	1.0
Obesity	3 (14)	2 (15)	1 (25)	1.0
Alcohol abuse	1 (5)	1 (8)	0 (0)	1.0
Psychiatric disorders	9 (43)	6 (46)	3 (38)	1.0
Days from hospital admission to diagnosis, median (range)	3 (0–44)	1 (0–44)	3 (0–9)	0.29
Leukocytes at diagnosis, cells/nl, median (range)°	9.7 (3.1–26.4)	10.3 (3.8–21)	9.0 (3.1–26.4)	0.72
CRP at diagnosis, mg/L, median (range) ~	27 (3–495)	21 (3–83)	123 (3.0–495)	0.09
Lactate at diagnosis, mmol/L, median (range)*	1.3 (0.6–4.8)	1.5 (0.6–4.8)	1.1 (1.0–1.3)	0.41
Dilatation of colon in cm, median (range) ~	9 (7–12)	9 (7–12)	9.5 (8–12)	0.32
Length of stay on ICU, days, median (range)	4 (0–22)	4 (0–6)	5 (2–22)	0.11
Readmission on ICU, *n* (%)	6 (29)	1 (8)	5 (63)	0.01
Length of hospital stay, days, median (range)	10 (4–78)	5 (4–78)	27 (10–66)	0.002
90-day mortality, *n* (%)	3 (21)	0 (0)	3 (38)	0.21

*CTx*, chemotherapy; *CAD*, coronary artery disease; *ICU*, intensive care unit; *PAD*, peripheral artery disease; *CRP*, c-reactive protein; °missing for 2 patients, ~ missing for 6 patients, ^#^missing for 16 patients,*missing for 10 patients

**Table 3:** Logistic regression analysis predicting the lack of therapeutic success in patients receiving upfront conservative management

	Univariate		
	OR	CI	*p* value
Age ≥ 65 years	4.4	0.4–47.0	0.22
Gender female	0.476	0.04–5.6	0.56
Primary reason for admission			
Trauma	5.5	0.71–42.6	0.10
Abdominal focus	0.389	0.06–2.7	0.34
Oncologic reason (CTx, etc.)	1.7	0.09–31.9	0.72
Internal medicine	0.0	0	1.00
Cardiac/vascular	0.786	0.06–10.4	0.86
Neurological/psychiatric	0.0	0	1.00
Comorbidities	0.0	0	1.00
Arterial hypertension	0.96	0.16–5.9	0.97
CAD	3.3	0.41–26.4	0.26
PAD	0.0	0	1.00
Atrial fibrillation	1.7	0.09–31.9	0.72
Stroke	0.0	0	1.0
Cancer	2.3	0.4–13.9	0.38
Previous abdominal surgery	1.1	0.142–8.7	0.92
Chronic kidney disease	0.0	0	1.0
Pulmonary disorders	0.960	0.2–5.9	0.97
Diabetes	1.7	0.09–31.9	0.72
Obesity	0.8	0.06–10.4	0.86
Alcohol abuse	0.0	0	1.00
Psychiatric disorders	0.7	0.1–4.2	0.7
Colon dilatation ≥ 9 cm	4	0.3–49.6	0.28
Colon dilatation ≥ 10 cm	1.25	0.158–9.9	0.83
Colon dilatation ≥ 11 cm	4	0.3–58.6	0.31
Leukocytosis ≥ 10 cells/nl at diagnosis	0.72	0.1–4.6	0.73
CRP ≥ 100 mg/L at diagnosis	0.0	0	1.0
Lactate ≥ 2 mmol/L at diagnosis	0.0	0	1.0

*CTx*, chemotherapy; *CAD*, coronary artery disease; *PAD*, peripheral artery disease; *CRP*, c-reactive protein

### Endoscopic management

In patients who underwent endoscopic treatment, a decompression tube was placed for 19 patients (76%), and in 10 patients (40%), a re-endoscopy was necessary. Similar to the conservative treatment group, 8 patients (32%) could not be treated successfully by endoscopy and were in need to undergo surgery due to the lack of endoscopic success (*n* = 1) or the occurrence of complications (*n* = 7). Complications included ischemia (*n* = 1), colonic perforation (*n* = 5), or both (*n* = 1).

Comparing patients who were and were not treated successfully by endoscopy (Table 4) showed that patients with therapeutic failure had a significantly higher diameter of colon dilatation (*p* = 0.001). Performing a logistic regression analysis identified a colon diameter ≥ 11 cm being the only factor to predict therapeutic failure of endoscopic treatment for Ogilvie’s syndrome (Table 5).

**Table 4:** Characteristics of patients with and without therapeutic success in receiving upfront endoscopic management

	Total	Therapeutic success	No therapeutic success	*p* value
	*n* = 25 (100%)	*n* = 17 (68%)	*n* = 8 (32%)	
Age in years, median (range)	64 (34–86)	67 (34–86)	62 (45–71)	0.35
Gender, *n* (%)				
Male	19 (76)	13 (76)	6 (75)	1.0
Female	6 (24)	4 (24)	2 (25)	
Primary reason for admission, *n* (%)				
Trauma	5 (20)	3 (18)	2 (25)	1.0
Abdominal focus	7 (28)	5 (29)	2 (25)	1.0
Oncologic reason (CTx, etc.)	3 (12)	3 (18)	0 (0)	0.53
Internal medicine	3 (12)	2 (12)	1 (12)	1.0
Cardiac/vascular	6 (24)	3 (18)	3 (38)	0.34
Neurological/psychiatric	1 (4)	1 (6)	0 (0)	1.0
Comorbidities, *n* (%)	23 (92)	17 (100)	6 (75)	0.09
Arterial hypertension	8 (32)	7 (41)	1 (12)	0.21
CAD	5 (20)	4 (24)	1 (12)	0.64
PAD	2 (8)	1 (6)	1 (12)	1.0
Atrial fibrillation	3 (12)	1 (6)	2 (25)	0.23
Stroke	4 (16)	2 (12)	2 (25)	0.57
Cancer	10 (40)	9 (53)	1 (12)	0.09
Previous abdominal surgery	9 (36)	7 (41)	2 (25)	0.66
Chronic kidney disease	8 (32)	6 (35)	2 (25)	0.68
Pulmonary disorders	7 (28)	4 (24)	3 (38)	0.64
Diabetes	3 (12)	3 (18)	0 (0)	0.53
Obesity	3 (12)	1 (6)	2 (25)	0.23
Alcohol abuse	3 (12)	3 (18)	0 (0)	0.53
Psychiatric disorders	4 (16)	3 (18)	1 (12)	1.0
Days from hospital admission to diagnosis, median (range)	6 (0–21)	5 (0–21)	6 (1–9)	0.97
Leukocytes at diagnosis, cells/nl, median (range)§	14.1 (7.5–35.6)	13.7 (7.5–35.6)	16.1 (7.7–22.6)	0.70
CRP at diagnosis, mg/L, median (range)*	150 (4–312)	93 (4–312)	160 (116–246)	0.60
Lactate at diagnosis, mmol/L, median (range)°	1.6 (0.7–3.8)	1.6 (0.7–3.8)	1.4 (0.9–1.9)	0.34
Dilatation of colon in cm, median (range) ~	9 (6–13)	9 (6–11)	12 (10–13)	0.001
Length of stay on ICU, days, median (range)	6 (0–49)	5 (0–49)	8 (1–28)	0.83
Readmission on ICU, *n* (%)	6 (24)	3 (18)	3 (38)	0.34
Length of hospital stay, days, median (range)	29 (4–71)	27 (4–71)	45 (26–64)	0.09
90-day mortality, *n* (%)	3 (19)	2 (12)	1 (12)	1.0

*CTx*, chemotherapy; *CAD*, coronary artery disease; *ICU*, intensive care unit; *PAD*, peripheral artery disease; *CRP*, c-reactive protein; §missing for 3 patients, *missing for 12 patients, #missing for 20 patients, °missing for 7 patients, ~ missing for 4 patients

**Table 5:** Logistic regression analysis predicting the lack of therapeutic success in patients receiving upfront endoscopic management

	Univariate		
	OR	CI	*p* value
Age ≥ 65 years	0.5	0.09–2.9	0.47
Gender female	1	0.2–7.6	0.94
Primary reason for admission			
Trauma	1.6	0.2–11.8	0.67
Abdominal focus	0.8	0.1–5.4	0.82
Oncologic reason (CTx, etc.)	0.0	0	1.0
Internal medicine	1.1	0.08–13.9	0.96
Cardiac/vascular	1.8	0.4–18.7	0.29
Neurological/psychiatric	0.0	0	1.0
Comorbidities	0.0	0	1.0
Arterial hypertension	0.2	0.02–2.1	0.18
CAD	0.5	0.04–4.9	0.53
PAD	2.3	0.1–41.9	0.58
Atrial fibrillation	5.3	0.4–70.2	0.20
Stroke	2.5	0.3–22	0.41
Cancer	0.1	0.01–1.4	0.08
Previous abdominal surgery	0.476	0.07–3.1	0.44
Chronic kidney disease	0.6	0.09–4	0.61
Pulmonary disorders	1,9	0.3–12	0.47
Diabetes	0.0	0	1.0
Obesity	5.3	0.4–70.2	0.20
Alcohol abuse	0.0	0	1.0
Psychiatric disorders	0.7	0.1–7.6	0.74
Colon dilation ≥ 9 cm	0.0	0	1.0
Colon dilatation ≥ 10 cm	0.0	0	1.0
Colon dilation ≥ 11 cm	28	1.9–394	0.01
Leukocytosis ≥ 10 cells/nl at diagnosis	0.8	0.1–6.3	0.85
CRP ≥ 100 mg/L at diagnosis	0.0	0	1.0
Lactate ≥ 2 mmol/L at diagnosis	0.0	0	1.0

*CTx*, chemotherapy; *CAD*, coronary artery disease; *PAD*, peripheral artery disease; *CRP*, c-reactive protein

### Surgical therapy

Reasons for upfront surgery were mainly acute abdomen (*n* = 12, 17%) followed by colonic perforation (*n* = 3, 12%) and sepsis (*n* = 3, 12%). Whereas for 20 patients (80%), an operative decompression (appendectomy, colotomy, ileo- or colostomy, or manual decompression) was performed; only 5 patients (20%) were in need of resection. A re-operation was necessary for 3 patients (12%) because of ischemia. Postoperative complications occurred in 84% of patients (*n* = 22) with a major complication (Dindo-Clavien classification ≥ IIIa) in 72% of patients (*n* = 19). Ninety-day mortality was 21% (*n* = 5).

### Overall survival

**Fig. 1:**
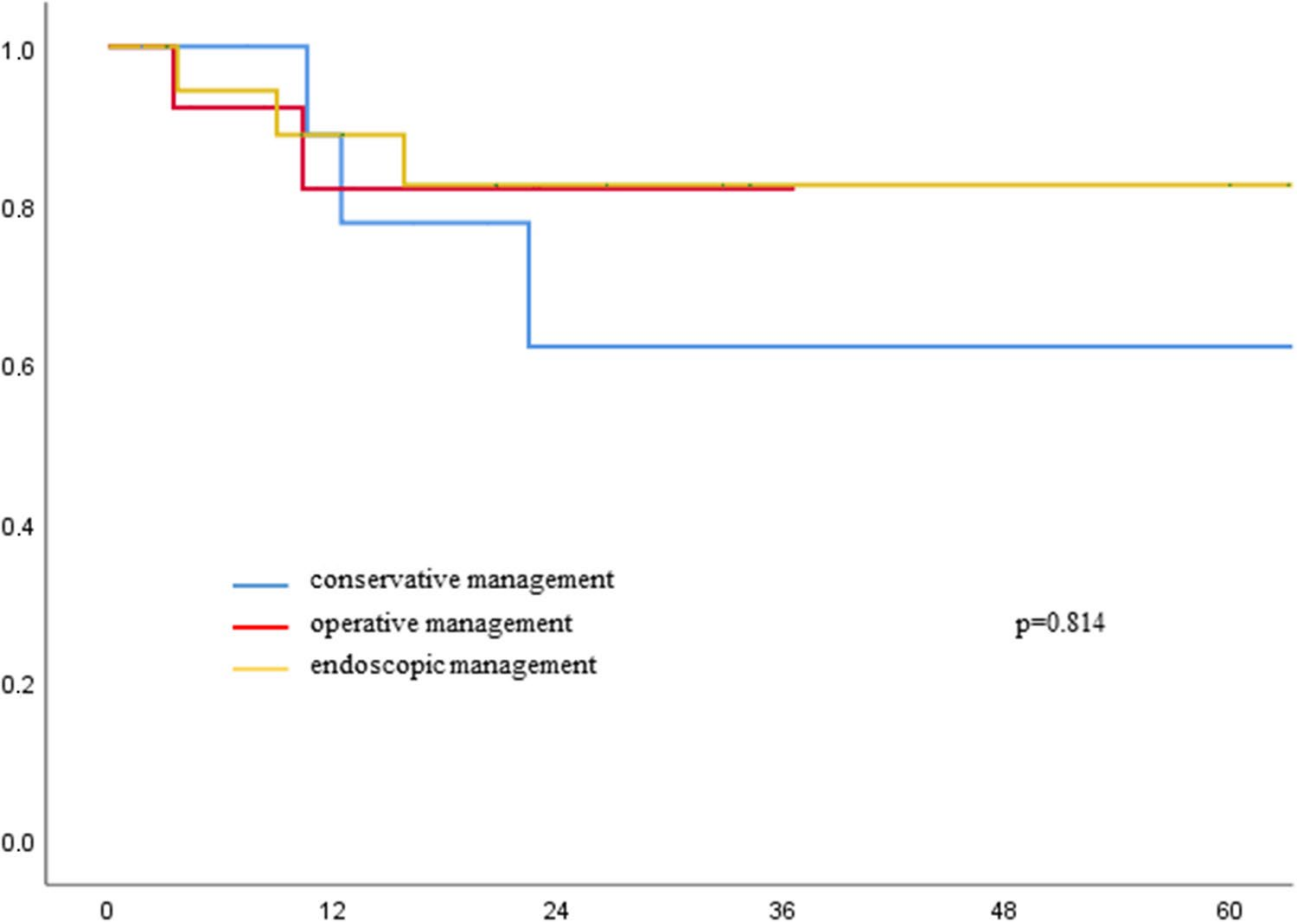
Overall survival of patients with Ogilvie Syndrome and different therapeutic strategies

**Fig. 2:**
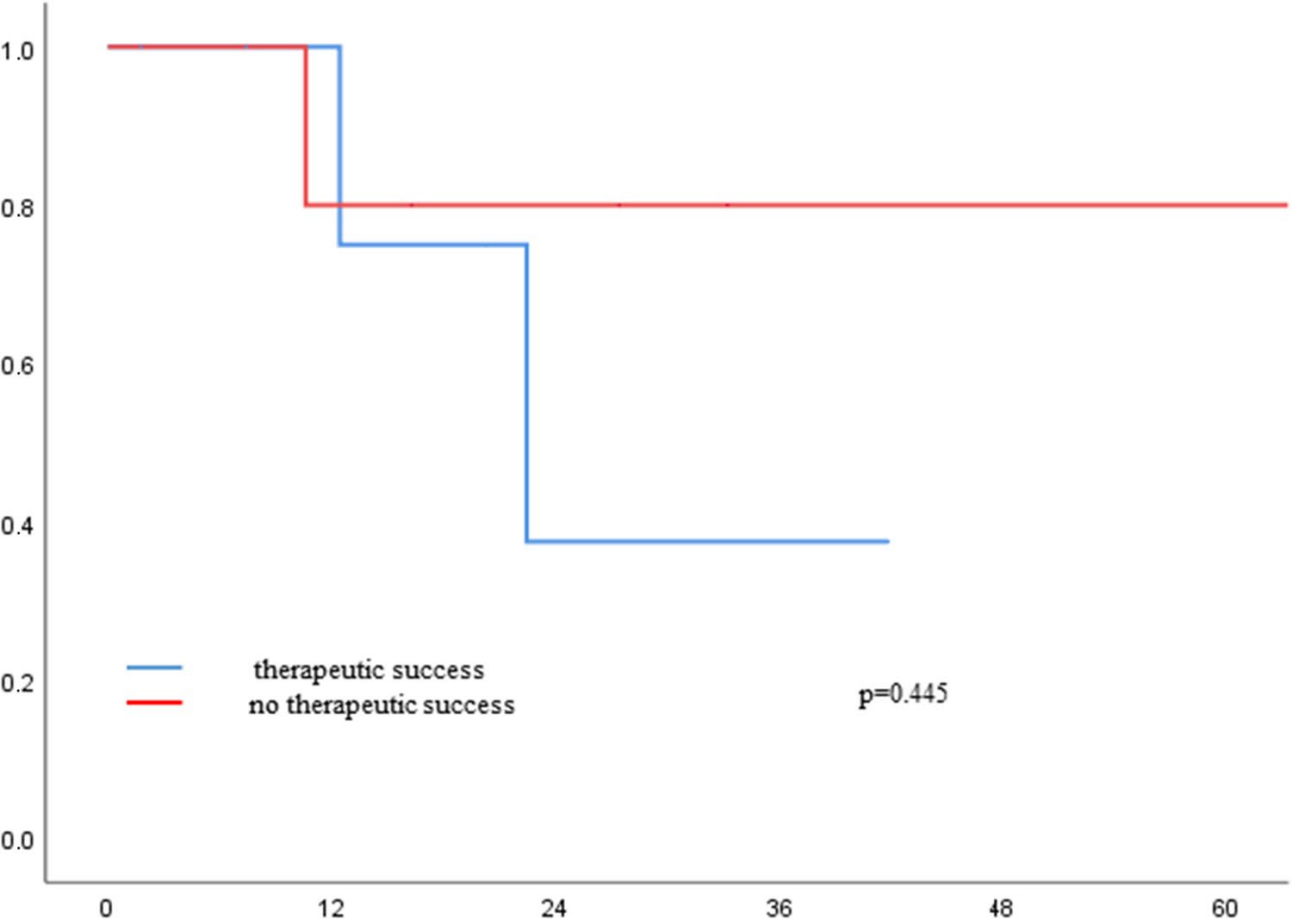
Overall survival of patients with conservative management

**Fig. 3:**
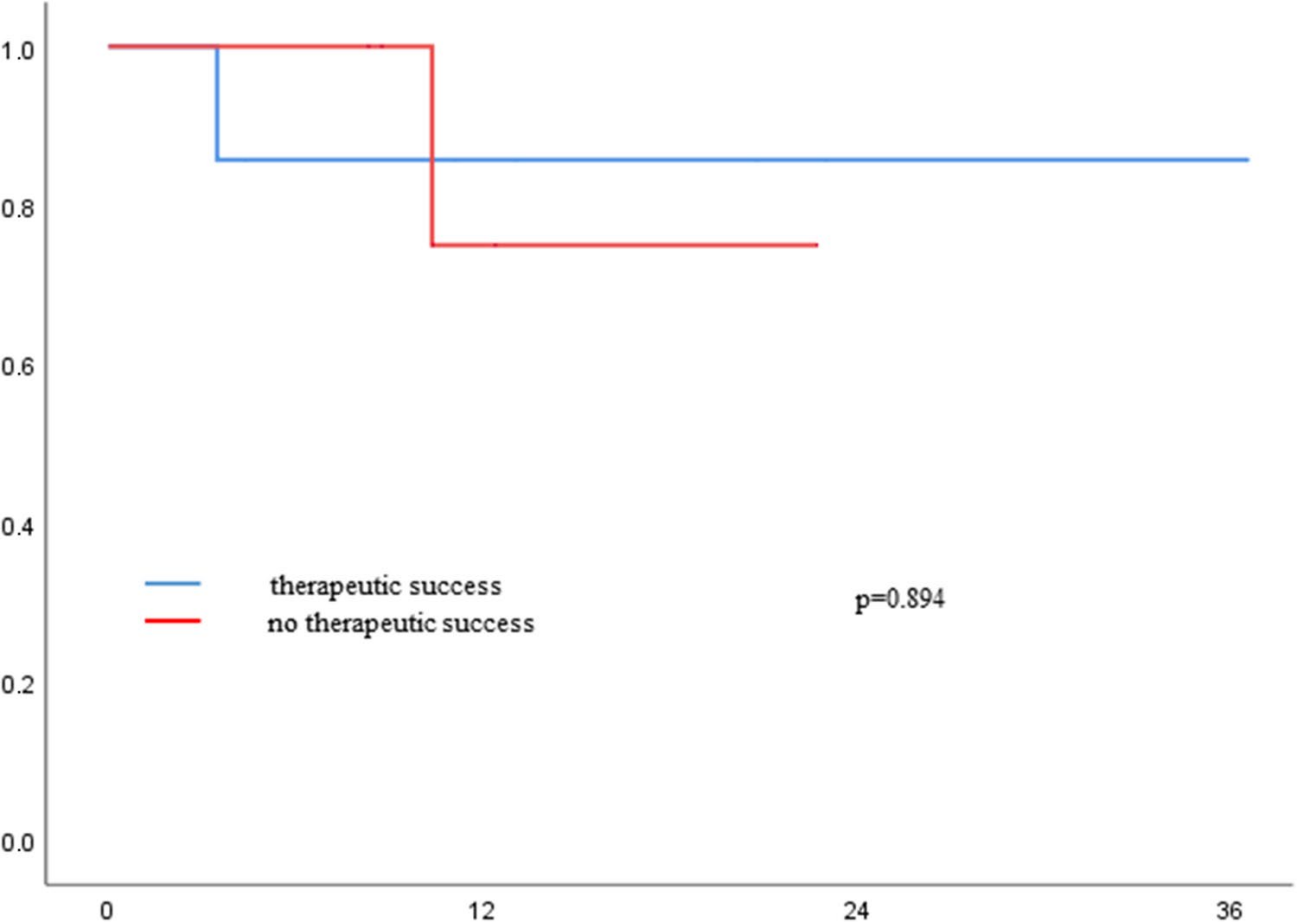
Overall survival of patients with endoscopic management

Mean overall survival was 22 months and comparable between the groups (*p* = 0.81; Fig. 1). Also for patients who were and were not treated successfully by conservative or endoscopic treatment, there was no difference in OS (conservative success vs failure *p* = 0.45; endoscopic success vs failure *p* = 0.89; Figs. 2 and 3).

## Discussion

This study shows that in patients with Ogilvie’s syndrome treatment failure is a quite common risk no matter if conservative treatment or endoscopy was the preferred therapeutic strategy. About one-third of patients in the conservative group had to proceed to endoscopy and/or surgery. Similarly, also about one-third of patients in the endoscopic group was in need to undergo surgery. However, the only factor to predict treatment failure was a colon diameter ≥ 11 cm in patients who were treated endoscopically.

Usually, surgery is the last step to take in patients with Ogilvie’s syndrome except they present with colonic perforation, ischemia, peritonitis, or sepsis. In this study, this was the case for 35% of the study population (25 patients) at the time of diagnosis of Ogilvie’s syndrome and these patients subsequently underwent upfront surgery. However, these patients suffered a high postoperative morbidity with 72% (*n* = 19) of patients having major complications with sepsis/septic shock, respiratory insufficiency, and burst abdomen occurring most often in our cohort. Therefore, the identified cutoff parameter of colonic dilatation of 11 cm might be appropriate to first lower the incidence of colonic perforation, ischemia, and sepsis and secondly to also reduce postoperative complications if patients are referred to surgery at an earlier stage instead to proceed with endoscopy. Similarly, the guidelines of the American Society for Gastrointestinal Endoscopy [4] recommend a caecal diameter > 12 cm to refer patients to surgery as otherwise the risk of perforation was significantly increased in a study by Vanek et al. [3]. Other risk factors described to predict endoscopic failure were female gender, emergent admission and metastatic cancer, and COPD as comorbidities [4]. Although almost all of the patients of our study population had severe comorbidities, this study could not identify any comorbidity to predict therapeutic failure. Regarding the decision to perform surgery for the treatment of patients with Ogilvie’s syndrome, the patients’ clinical presentation could clearly impact the decision-making. Recommending surgery for an asymptomatic patient with a colonic dilatation of 11 cm or more is a really challenging decision. However, our study clearly presented, that even these asymptomatic patients are at high risk for the development of colonic ischemia and perforation. In our study population, over 50% of the patients with a colonic dilation of 11 cm or more who were primarily treated conservatively or by endoscopy did not respond to the therapy and had consequently undergone surgery. Therefore, we clearly recommend surgery as the therapy to choose in asymptomatic patients with a colonic dilation ≥ 11 cm, especially if patients have further risk factors such as immunosuppressive therapy.

Not only for endoscopic but also for conservative management, the failure rates are quite high with over 30%. However, there must be noted that the definitions of treatment failure are different for the two groups. In patients with conservative treatment failure, only 28.5% (*n* = 6) of patients had to proceed to surgery whereas all of the patients with endoscopic treatment failure underwent surgery. Therefore, conservative treatment might be the first therapy of choice because a significant number of patients responded to this treatment with the welcome association of a shorter length of stay. These results are supported by the newest guidelines who recommend medical therapy before proceeding with endoscopy [4]. However, if conservative therapy or endoscopy is the better treatment strategy for patients with Ogilvie’s syndrome, it was examined by two studies which both concluded endoscopy to be superior to medical therapy [7, 8]. Endoscopy was not only more effective compared to the medical therapy [7, 8] but also the chance of avoiding a second treatment modality was higher [7]. A review examining this matter found the endoscopic and medical treatment to be comparable [9]. No matter, if conservative or endoscopic treatment is chosen for upfront therapy, a close and interdisciplinary patient observation is necessary for our opinion not to miss the right time point for a more aggressive treatment strategy. This might be necessary if the colonic diameter is not decreasing, clinical symptoms or lab results are worsening.

This study has some limitations. First, the retrospective, single-institutional design with a selective cohort of patients leads to the risk of selection bias. Therefore, it is possible that patients with more severe comorbidities might have been treated more aggressively compared to healthier patients. Second, the small study population in a long study period of 17 years in which therapeutic strategies might have changed. However, Ogilvie’s syndrome is a quite uncommon disease; therefore, only few publications with a higher number of patients exist.

## Conclusion

Conservative and endoscopic treatment strategies fail in over 30% of patients. As treatment failure increases the severe risk of colonic perforation and ischemia a cutoff diameter of colon dilatation of 11 cm might be appropriate to refer patients to surgery and not to proceed with endoscopy.

## Data Availability

The data that support the findings of this study are available on request from the corresponding author after approval of the institution’s ethics committee. The data are not publicly available due to privacy or ethical restrictions.
